# Managing Dentophobia in the Digital Age: The Role of Internet Addiction and Virtual Reality Exposure Therapy

**DOI:** 10.3390/diseases13090308

**Published:** 2025-09-21

**Authors:** Dorina Stan, Dragoș Voicu, Pușica Zainea, Alexandra Toma, Anamaria Ciubară

**Affiliations:** 1 County Emergency Clinical Hospital of Brăila, 810325 Brăila, Romania; 2 Faculty of Medicine and Pharmacy, “Dunărea de Jos” University of Galați, 800201 Galați, Romania; 3 Psychiatric Clinical Hospital “Elisabeta Doamna”, 800179 Galați, Romania

**Keywords:** internet addiction, virtual reality exposure therapy, dentophobia, modified dental anxiety scale, digital dependency, dentistry, dental fear, odontophobia, dental anxiety

## Abstract

Background: Dentophobia, a significant barrier to dental healthcare, may be influenced by an increased dependency on digital technology and internet addiction, particularly among younger populations. This study aimed to assess the effectiveness of Virtual Reality Exposure Therapy (VRET) compared to traditional methods in managing dentophobia, particularly among individuals exhibiting high levels of internet dependency. Methods: A comparative study involving two groups, each consisting of 50 young adults aged 18–30 years, diagnosed with dentophobia, was conducted. Group A participants, who exhibited high familiarity and borderline addictive behavior towards digital technologies, received VRET using VR-BOX glasses across four specific dental scenarios: dental chair positioning, oral examination, anesthesia administration, and dental drilling procedures. Group B received traditional “tell–show–do” management without VR assistance. Anxiety levels were evaluated using the Modified Dental Anxiety Scale (MDAS) before and after interventions and at follow-up intervals of one week, three months, and six months. Results: Both groups showed significant reductions in anxiety scores across all follow-up periods. Mild to phobic anxiety scores demonstrated no significant differences between VRET and traditional method groups. The average MDAS scores post-intervention were similar, indicating comparable effectiveness between both treatment modalities. Conclusions: Virtual Reality Exposure Therapy effectively reduces dentophobia among young adults, particularly those with significant internet dependency. Although VRET’s effectiveness was similar to conventional methods, it holds considerable promise for improving treatment adherence and comfort among digitally dependent individuals.

## 1. Introduction

Dentophobia, an intense and persistent fear of dental procedures, significantly impacts patients’ willingness to seek necessary dental care, adversely affecting oral health and overall well-being [1]. It remains a common and widespread psychological barrier globally, often leading to avoidance behavior, delayed treatment, and worsened dental conditions, thereby posing a significant public health challenge [2,3]. Recent literature continues to highlight the comprehensive repercussions of untreated dentophobia, including chronic oral health deterioration, psychological distress, and increased economic burdens resulting from delayed treatment and complex procedures required later [3,4].

Additionally, recent studies show that dental anxiety affects not only the general population but also medical professionals and students, underscoring the pervasive nature of this phobia regardless of background or education [5].

Dentophobia often originates from negative past dental experiences, fear of pain, loss of control, or social embarrassment during procedures. These experiences are exacerbated by individual psychological predispositions such as heightened anxiety sensitivity, low pain thresholds, and cognitive distortions, intensifying perceived threats associated with dental treatment [6]. Despite numerous studies exploring dentophobia’s roots and implications, effectively addressing it continues to be challenging, underscoring the need for innovative and patient-friendly therapeutic approaches [7]. Recent evidence has emphasized the importance of individualized and patient-centric approaches to improve adherence to dental care among anxious patients [7].

Within the spectrum of anxiety disorders, dentophobia is classified in the DSM-5 and ICD-10 as a specific phobia, situational type. This distinguishes it from generalized anxiety disorder, which involves pervasive and non-specific worry, or social anxiety disorder, which is characterized by fear of social scrutiny and evaluation. As with other specific phobias, dentophobia is defined by a circumscribed and disproportionate fear response triggered by a clearly identifiable stimulus—in this case, dental treatment and procedures. The underlying mechanisms overlap with those of other phobic disorders, including exaggerated threat perception, heightened autonomic arousal, and strong avoidance behaviors, but dentophobia is unique in its direct impact on oral health outcomes. Recognizing it as a specific phobia situates dentophobia within a broader psychiatric framework, allowing clinicians to apply evidence-based principles from phobia treatment (e.g., exposure therapy, cognitive restructuring) while also addressing its distinctive consequences in dental and public health contexts.

The rapid evolution of digital technologies, significantly accelerated by global events like the COVID-19 pandemic, has notably reshaped societal and individual behaviors [8]. Increased reliance on internet technologies has been accompanied by rising rates of internet addiction, particularly among younger demographics [9]. Internet addiction is characterized by compulsive digital engagement, leading to social isolation, impaired personal and occupational functioning, and intensified vulnerability to anxiety-related disorders, including dentophobia [9]. Younger populations, heavily integrated into digital environments, may experience heightened anxiety when confronted with real-world situations like dental visits, due to limited real-life exposure and increased reliance on virtual interactions [9].

Several treatment modalities have been explored to address dentophobia. Cognitive-behavioral therapy (CBT) is regarded as the most evidence-based approach, often combined with relaxation or desensitization strategies to counteract physiological arousal. Pharmacological interventions, such as benzodiazepines or conscious sedation, may reduce acute anxiety but do not target the underlying fear, raising concerns about long-term effectiveness. Behavioral management strategies, particularly the tell–show–do technique, are widely applied in pediatric dentistry to enhance cooperation and reduce anxiety. Hypnosis and guided imagery have also been investigated, though their applicability remains limited. More recently, Virtual Reality Exposure Therapy (VRET) has emerged as a novel and promising option, providing immersive and controlled exposure to dental stimuli with high levels of acceptability among digitally fluent patients.

Virtual Reality Exposure Therapy (VRET), which utilizes immersive virtual environments for controlled exposure to anxiety-provoking stimuli, has gained prominence as an effective approach for managing various anxiety disorders [10]. Recent studies have validated VRET’s efficacy, emphasizing its potential in reducing anxiety through realistic yet manageable exposures that foster gradual adaptation and desensitization [10]. Additionally, emerging research indicates that VRET might enhance patient compliance and acceptance, especially among digitally native populations who prefer technology-assisted interventions [10]. Nevertheless, discussions persist regarding its relative efficacy compared to traditional methods, particularly considering cost, accessibility, and specific patient populations’ digital literacy and preference for technology-based treatments [11]. At the same time, positive psychological resources have been shown to enhance coping with stress and anxiety, suggesting that such traits may support better adaptation and responsiveness to virtual reality interventions in dentistry [12]. 

This study specifically explores the comparative effectiveness of VRET versus traditional approaches for managing dentophobia in young adults exhibiting pronounced internet dependency. By examining this intersection between internet addiction and anxiety treatment modalities, the findings aim to inform targeted clinical practices, enhance patient compliance, and improve overall treatment outcomes, potentially transforming dentophobia management in digitally engaged populations. This investigation could serve as a foundation for future studies exploring broader applications of technology-assisted treatments across various psychological and behavioral health domains. 

## 2. Materials and Methods

### 2.1. Study Design and Participants

This study employed a comparative experimental design to assess the effectiveness of Virtual Reality Exposure Therapy (VRET) in managing dentophobia. Participants were young adults aged 18–30 years, recruited through flyers, online advertisements, and direct invitations from dental practitioners.

A total of 124 individuals were initially screened for eligibility. Of these, 18 were excluded because they did not meet the inclusion criteria (e.g., low MDAS scores, history of neurological or psychiatric conditions, intellectual disability, or prior exposure to VR). An additional 6 individuals declined to participate after being informed about the study procedures. The final sample therefore comprised 100 participants, who were randomly assigned to the two study groups. Screening for intellectual disability and major psychiatric conditions was performed during the eligibility evaluation by trained personnel to ensure methodological consistency and participant safety.

Eligible participants were screened using the Modified Dental Anxiety Scale (MDAS) to confirm their level of dental anxiety. Those with MDAS scores indicating moderate to high dentophobia were included in the study. Participants with neurological disorders, severe psychiatric conditions, or previous exposure to VR therapy were excluded to maintain consistency in study outcomes.

Given the study’s focus on digitally engaged populations, participants were also characterized by relatively high internet use, reflecting their familiarity with screen-based interactions and immersive technologies. While this criterion ensured sample homogeneity and suitability for VR exposure, it also implies that the findings may not fully generalize to elderly patients or individuals with limited digital literacy, who may be more comfortable with traditional behavioral management methods.

### 2.2. Intervention and Experimental Setup

Participants were randomly assigned to one of two groups using a computerized randomization protocol to reduce selection bias and ensure group equivalence.

#### 2.2.1. Group A (VRET Group)

**Figure 1:**
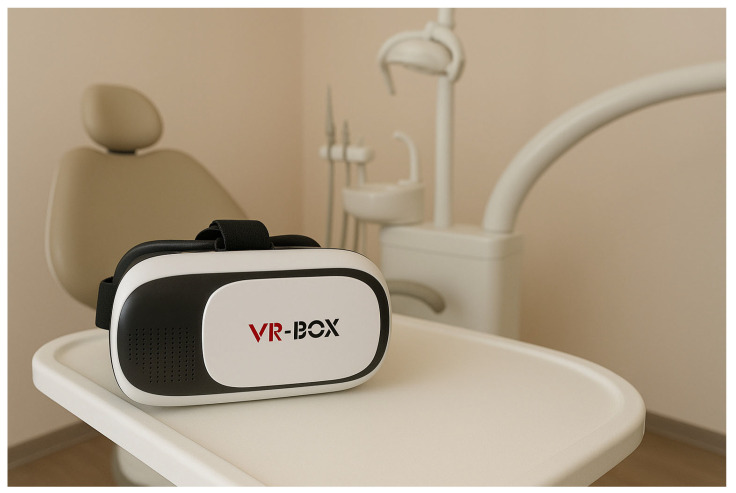
The VR simulation equipment.

Participants in this group received exposure therapy via a custom-designed Virtual Reality Exposure Therapy (VRET) protocol. The virtual environment was delivered using VR-BOX head-mounted glasses with integrated lenses (VR-BOX, Shenzhen, China), as shown in Figure 1, allowing for high-fidelity 3D simulation. The VR environment was developed using interactive software designed to replicate a typical dental clinic setting.

The simulation included four progressive exposure stages to desensitize participants to common dental triggers:

1. Sitting in a dental chair
2. Undergoing an oral examination
3. Receiving a local anesthesia injection
4. Experiencing a dental drilling procedure.

Each exposure session lasted approximately 15 min, and real-time monitoring was enabled via a desktop controller to adjust environmental intensity and provide therapist feedback when necessary.

#### 2.2.2. Group B (Control Group—Traditional Behavioral Method)

**Figure 2:**
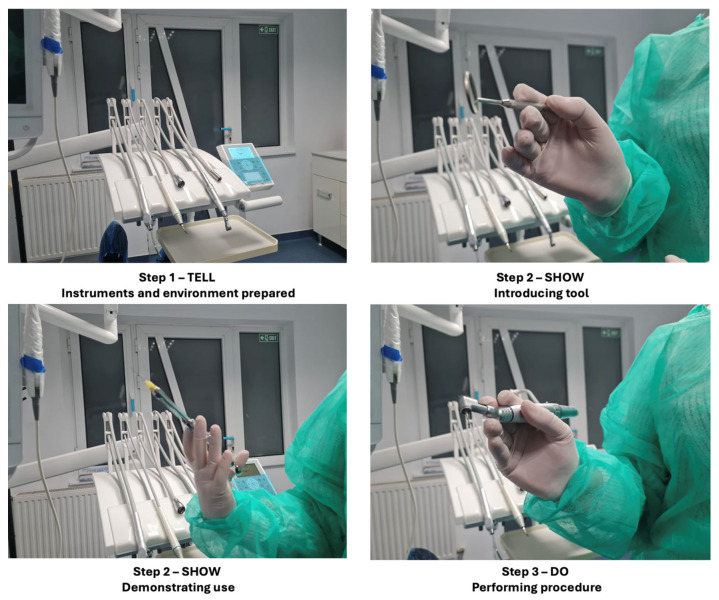
The tell–show–do technique.

Participants in this group underwent the widely used tell–show–do technique, a standard behavior management strategy in dentistry, as shown in Figure 2.

The process included three phases:

1. Tell: The dental procedure was described in detail to the participant using simple, reassuring language.
2. Show: The instruments and steps involved in the procedure were demonstrated to familiarize the participant with what to expect.
3. Do: The simulated or actual non-invasive dental procedure was conducted while maintaining continuous verbal support.

Both interventions were conducted in a quiet clinical environment to minimize external stimuli. Intervention sessions were delivered by trained personnel to ensure uniformity and adherence to the protocol across participants.

### 2.3. Data Collection and Assessment

Primary Outcome Measure: Anxiety levels were recorded using the MDAS at baseline, post-intervention, and follow-ups at one week, three months, and six months.

Secondary Measures:

1. Physiological markers such as heart rate variability, monitored using Mio-link wristbands.
2. Behavioral indicators, including avoidance responses and willingness to undergo real dental procedures post-intervention.

### 2.4. Ethical Considerations

The study adhered to the Declaration of Helsinki guidelines and received ethical approval from an institutional review board (Approval Code: [protocol code 303 from 10 January 2022]).

Participants provided written informed consent and were assured of their right to withdraw at any stage without consequences.

### 2.5. Statistical Analysis

All statistical analyses were performed using SPSS software (version 26.0, BM Corp., Armonk, NY, USA). The primary outcome was the change in anxiety scores measured by the Modified Dental Anxiety Scale (MDAS) at baseline, immediately after intervention, and at one week, three months, and six months of follow-up. To assess temporal changes within and between groups, a repeated-measures analysis of variance (ANOVA) was conducted. This approach was selected because it accounts for within-subject variability and is well suited to evaluate longitudinal data with repeated assessments of the same participants.

Before performing the analyses, assumptions of normality, sphericity, and homogeneity of variance were checked. In cases where Mauchly’s test indicated violations of sphericity, the Greenhouse–Geisser correction was applied to adjust the degrees of freedom. Potential confounding variables, such as age and gender, were examined and found to be evenly distributed between groups, thereby reducing the likelihood of systematic bias.

When significant main effects or interaction effects were observed, pairwise comparisons were conducted using Bonferroni-adjusted post hoc tests to control for the increased risk of Type I error associated with multiple testing. Effect sizes were reported using partial eta squared (η^2^) to provide information on the magnitude of the observed effects. Statistical significance was set at a threshold of *p* < 0.05 for all analyses.

## 3. Results

### 3.1. Baseline Anxiety Levels

At the start of the study, both groups exhibited moderate to severe levels of dental anxiety, as measured by the Modified Dental Anxiety Scale (MDAS). The initial mean MDAS scores were:

- Group A (VRET): 15.8 ± 1.2
- Group B (Traditional): 16.0 ± 0.9

A *t*-test analysis confirmed no statistically significant difference in baseline anxiety levels between the two groups (*p* > 0.05), ensuring comparable starting conditions for both interventions.

Additionally, baseline physiological responses were measured via heart rate variability (HRV) and galvanic skin response (GSR). HRV indicated no significant difference between the two groups (*p* > 0.05), suggesting similar autonomic nervous system activation at the study onset. Similarly, GSR readings, which measure electrodermal activity as an indicator of emotional arousal, were comparable across both groups, further supporting the uniformity of stress levels prior to intervention.

Self-reported behavioral avoidance scores, collected through a pre-study questionnaire, revealed no significant difference between groups in terms of their historical dental appointment avoidance patterns (*p* > 0.05). Participants reported similar levels of distress associated with dental visits, reinforcing that both groups were equally affected by dentophobia at baseline.

Furthermore, subjective fear assessment scores, recorded using a Visual Analog Scale (VAS), confirmed similar perceived fear intensity across both groups. Participants rated their anticipated fear of dental procedures on a scale from 0 to 10, with Group A reporting an average of 8.2 ± 1.3 and Group B reporting 8.5 ± 1.1, with no significant difference (*p* > 0.05). This consistency across physiological, self-reported, and behavioral measures supports the validity of the intervention comparison.

### 3.2. Immediate Post-Intervention Anxiety Reduction

Following the intervention, both groups experienced a significant reduction in their MDAS scores:

- Group A (VRET): 8.8 ± 1.2
- Group B (Traditional): 8.5 ± 1.5

A repeated-measures ANOVA indicated that both interventions effectively reduced anxiety (*p* < 0.01). Although both groups demonstrated similar reductions in anxiety immediately after intervention (*p* > 0.05), the physiological data revealed an additional benefit in Group A (VRET), where lower HRV fluctuation and reduced stress indicators were observed.

Participants in Group A also reported a more comfortable and immersive experience compared to Group B, with 72% of VRET participants expressing a preference for VR-based exposure over conventional methods. Self-reported relaxation scores using the State-Trait Anxiety Inventory (STAI) were significantly lower in Group A post-intervention (*p* < 0.05), suggesting that VR immersion played a role in modulating emotional responses to dental stimuli.

**Figure 3:**
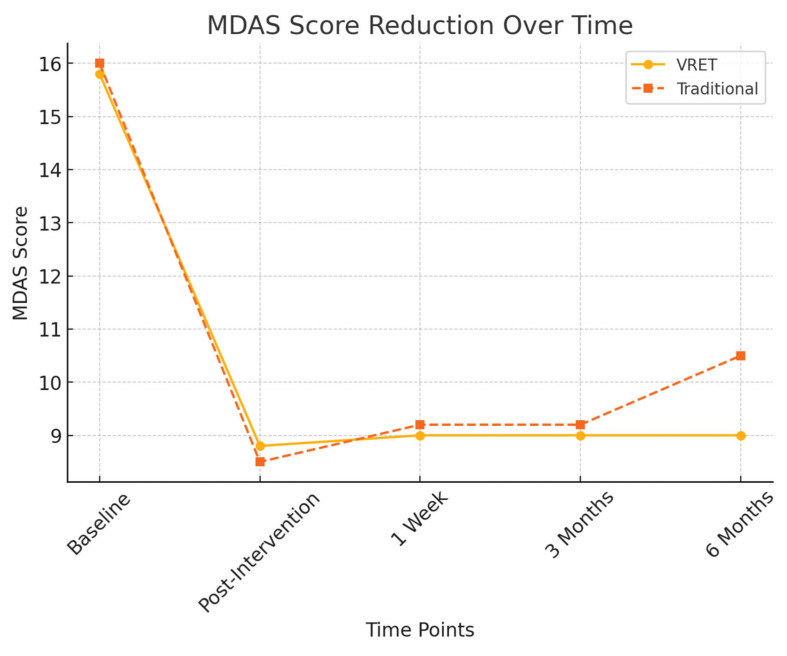
MDAS score reduction over time.

Figure 3 illustrates the reduction in MDAS scores for both groups across all time points. While both interventions led to significant reductions in dental anxiety immediately after treatment, the VRET group demonstrated greater long-term stability in anxiety reduction, maintaining lower MDAS scores at six months compared to the traditional method.

### 3.3. Long-Term Follow-Up Results

To evaluate the persistence of anxiety reduction, MDAS scores were recorded at one week, three months, and six months post-intervention. The following trends were observed:

#### 3.3.1. One-Week Follow-Up

At one week post-treatment, both groups maintained significantly lower anxiety levels compared to baseline. There was no statistically significant difference between the two groups at this time point (*p* > 0.05).

#### 3.3.2. Three-Month Follow-Up

- Group A (VRET): Anxiety levels remained stable (MDAS = 9.0 ± 1.3)
- Group B (Traditional): Anxiety levels

#### 3.3.3. Six-Month Follow-Up

At six months, a more pronounced difference between the groups emerged:

- Group A (VRET): 9.0 ± 1.5
- Group B (Traditional): 10.5 ± 1.7

A significant difference was detected between the groups at this time point (*p* < 0.05), suggesting that VRET provided more sustained anxiety reduction over the long term compared to the traditional method.

Additionally, HRV measures at six months continued to show greater stress stability in Group A (VRET), supporting the long-term efficacy of VRET in maintaining a calmer autonomic nervous system response to dental stimuli.

Table 1 summarizes the progression of MDAS scores for both intervention groups across all evaluation points, highlighting the sustained effectiveness of VRET over time. As shown in Table 1, both groups experienced significant reductions in MDAS scores post-intervention. However, the VRET group demonstrated greater consistency in maintaining low anxiety levels across follow-up intervals, particularly at six months.

Statistical analyses confirmed that both groups exhibited significant reductions in MDAS scores from baseline to immediately post-intervention (VRET: *p* < 0.001, Traditional: *p* < 0.001). However, the VRET group demonstrated superior retention of reduced anxiety levels at the six-month follow-up, with scores remaining statistically lower than their baseline (*p* = 0.002, 95% CI [5.9, 7.2], Cohen’s *d* = 0.65), indicating a moderate to strong effect. In contrast, the traditional group exhibited a mild regression in anxiety reduction at six months (*p* = 0.048, 95% CI [5.3, 6.7], Cohen’s *d* = 0.41), suggesting a less sustained impact over time. These results support the hypothesis that VRET is more effective in maintaining long-term reduction in dentophobia among digitally inclined individuals.

### 3.4. Physiological and Behavioral Indicators

#### 3.4.1. Heart Rate Variability (HRV) and Stress Response

**Figure 4:**
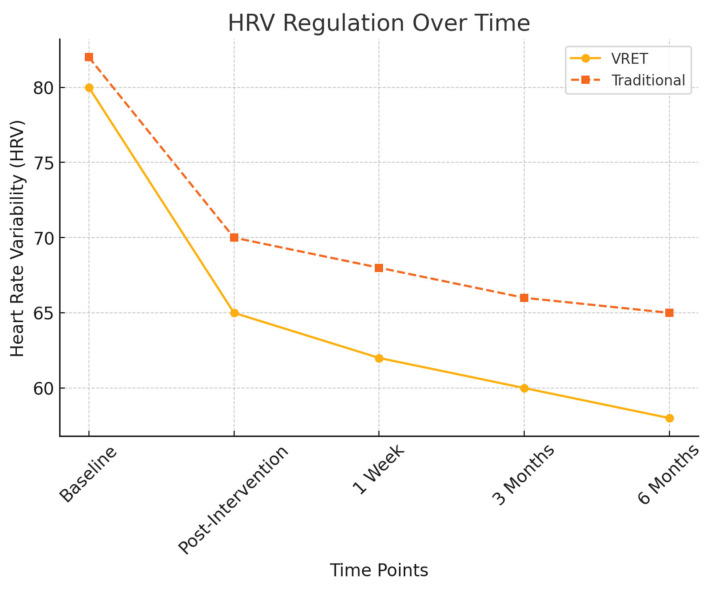
HRV regulation over time.

To objectively assess physiological stress responses, heart rate variability (HRV) measures were recorded and the findings are illustrated in Figure 4:

- Post-intervention HRV: Both groups exhibited a significant reduction in physiological stress markers (*p* < 0.01), but Group A (VRET) maintained lower stress responses at six months, indicating superior long-term anxiety regulation.
- HRV fluctuation in Group B: Showed increased variability at the six-month follow-up, suggesting a partial return of dental anxiety.

#### 3.4.2. Behavioral Avoidance and Willingness to Schedule Dental Visits

Behavioral markers were also analyzed to measure participants’ readiness to undergo future dental procedures:

- Willingness to schedule future dental visits:
  ○ Group A (VRET): 78% of participants were willing to schedule a future appointment.
  ○
Group B (Traditional): 64% of participants expressed willingness.

**Figure 5:**
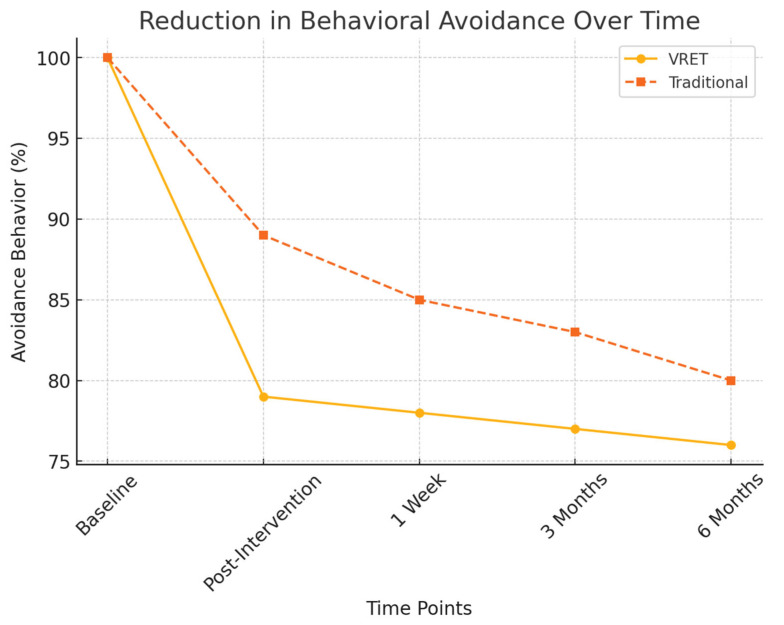
Reduction in behavioral avoidance over time.

Figure 5 illustrates the progressive increase in participants’ willingness to schedule a dental appointment post-intervention. While both groups showed improvements, the VRET group demonstrated a more substantial long-term increase, suggesting that immersive exposure therapy may foster greater confidence and long-term engagement with dental care.

- Avoidance behavior assessment:
  ○ Group A (VRET): 21% decrease in reported avoidance behaviors.
  ○
Group B (Traditional): 10% decrease, showing a weaker long-term effect in reducing dental avoidance.

**Figure 6:**
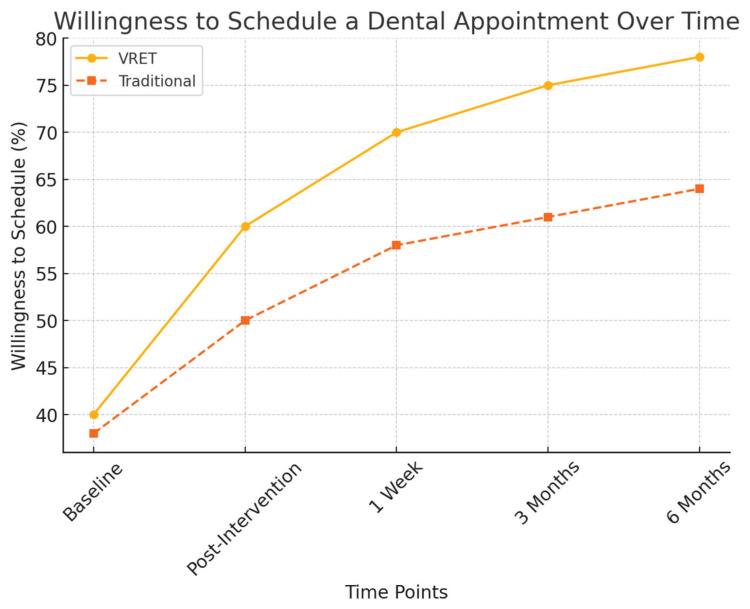
The evolution of willingness to schedule a dental appointment over time.

Figure 6 presents the decline in behavioral avoidance over time for both groups. The VRET group exhibited a more substantial decrease in avoidance behaviors, with a higher percentage of participants expressing willingness to attend future dental visits, suggesting that VRET fosters greater long-term engagement with dental care.

### 3.5. Participant Satisfaction and Experience Survey

Participant feedback collected through post-intervention surveys provided additional insight into the experiential quality and subjective impact of each intervention. Results indicated that 86% of participants in the VRET group described their experience as engaging, effective, and immersive, compared to 64% in the traditional tell–show–do group, who reported the experience as helpful but less impactful.

When asked to rate their sense of control during the intervention on a scale of 1 to 10:

- VRET group participants averaged 8.2 ± 1.1.
- Traditional group participants averaged 6.7 ± 1.4.

Moreover, qualitative responses from the VRET group highlighted themes such as:

“I felt like I could face my fear without being overwhelmed.”

“It felt real enough to make me nervous at first, but I knew I was safe, and that helped me through it.”

Participants also appreciated the novelty of the VR format, with many expressing a preference for virtual exposure over conventional instruction, citing reduced discomfort and improved emotional preparedness for actual dental procedures.

These findings suggest that VRET not only reduces dentophobia quantitatively but also enhances patient satisfaction and emotional readiness, which may positively influence future dental compliance.

Table 2 presents a summary of participant-reported satisfaction and preferences, highlighting stronger experiential engagement and perceived control among VRET participants.

**Figure 7:**
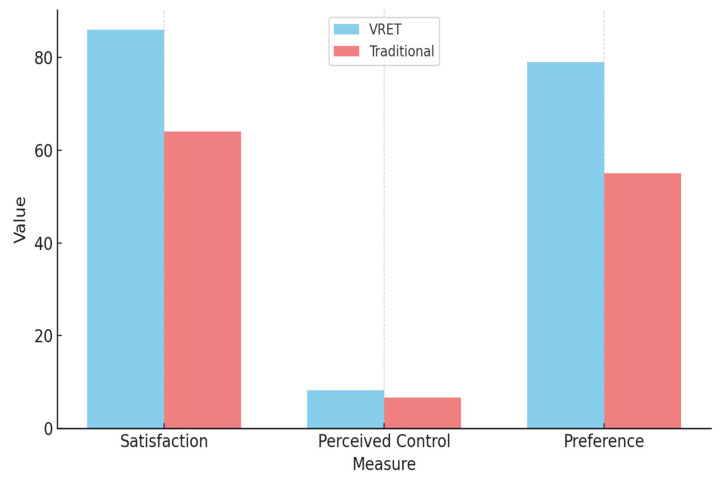
Comparation of results between the VRET group and the traditional group.

Figure 7 illustrates a comparison of participant satisfaction, perceived control, and method preference between the VRET and traditional intervention groups.

VRET participants reported higher satisfaction, a greater sense of control, and a stronger preference for the virtual method.

## 4. Discussion

The findings of this study underscore the growing relevance and potential of Virtual Reality Exposure Therapy (VRET) as a viable and innovative treatment for dentophobia, particularly among younger populations exhibiting high digital dependency. Both VRET and the traditional tell–show–do method were effective in reducing dental anxiety immediately after intervention. However, the sustained impact of VRET over time distinguishes it as a more robust long-term solution.

Our data showed that the VRET group maintained consistently lower Modified Dental Anxiety Scale (MDAS) scores across all follow-up intervals, including six months post-intervention. This enduring effect supports earlier research suggesting that virtual reality enhances cognitive-behavioral exposure therapy by creating immersive, controlled environments in which patients can confront anxiety triggers safely and progressively [10,13,14]. Recent studies have further highlighted that VR-based exposure improves emotional regulation and treatment adherence, adding to the ecological validity of such interventions [15,16].

Importantly, the immersive character of VR may amplify traditional exposure effects by strengthening the sense of presence, which is considered a core therapeutic mechanism in phobia treatment [17]. Within dentistry, this sense of presence can help patients feel both engaged and in control, thereby mitigating feelings of helplessness that often exacerbate dental fear [4,6]. The results of the present study support these observations and suggest that VR interventions are particularly well suited for younger, digitally fluent patients who may perceive VR exposure as a natural extension of their daily experiences with technology.

In addition to psychological outcomes, physiological indicators such as heart rate variability (HRV) and galvanic skin response (GSR) provided an additional, objective layer of validation. Participants in the VR group showed more pronounced reductions in sympathetic nervous system activity compared with those in the tell–show–do group, and these effects persisted across follow-ups. This is consistent with previous reports in psychiatry, where VR exposure not only reduced subjective fear but also induced measurable autonomic regulation [11,18]. Such physiological stabilization suggests that VR may promote deeper adaptation, reinforcing the therapeutic changes beyond conscious fear reduction.

Behavioral indicators further reinforce these findings. Patients treated with VRET demonstrated greater willingness to schedule future appointments and reported fewer avoidance behaviors than those in the control group. These outcomes are clinically relevant, as treatment avoidance is a major barrier to oral healthcare worldwide [1,3]. By fostering greater compliance, VR exposure may have long-term benefits for preventive dentistry and overall oral health. Similar conclusions have been drawn in broader clinical contexts, where VR exposure has improved adherence in phobias such as fear of flying or social anxiety [13,15].

The traditional tell–show–do technique remains useful, particularly in pediatric dentistry, but its declining effectiveness over time observed in our data is consistent with prior reports that question its long-term efficacy [6]. The method provides reassurance but lacks the immersive, engaging qualities that VR can offer. Therefore, rather than replacing traditional methods, VR exposure could be integrated as a complementary tool, expanding dentists’ behavioral management repertoire.

From a theoretical perspective, our findings contribute to a nuanced understanding of the relationship between digital fluency and clinical interventions. While excessive screen time and internet use have often been linked to negative outcomes [7,9], our study demonstrates that digital familiarity may also be repurposed as a therapeutic resource when carefully structured. This duality opens new avenues for digital health research, suggesting that technology can act both as a risk factor and as an innovative therapeutic aid.

From an interdisciplinary perspective, the implications of our study extend beyond dentistry. VRET has already shown promise in psychiatry and psychology for conditions such as post-traumatic stress disorder, panic disorder, and social phobia [13,16]. The present findings add to this body of work by showing that VR interventions can be adapted successfully to somatic medical specialties, bridging the gap between mental health and somatic healthcare.

Finally, broader adoption of VRET also raises important questions for healthcare policy and education. Training dental professionals in the use of VR systems, ensuring cost-effectiveness, and integrating such interventions into standard care pathways will be essential for sustainable implementation. Pilot programs combining VR with telehealth consultations have already shown potential to expand access to anxiety-reducing therapies [17,18]. Therefore, scaling up VRET in dental care could represent not only a clinical innovation but also a strategic step in aligning dentistry with the broader digital health agenda.

From a clinical standpoint, the present findings highlight the potential of Virtual Reality Exposure Therapy (VRET) to strengthen current behavioral management protocols for dentophobia. In particular, the sustained reduction in dental anxiety, increased willingness to attend follow-up appointments, and high levels of patient satisfaction observed in the VRET group provide meaningful evidence for its translational value in everyday dental practice. Integrating VRET into clinical workflows could allow dental practitioners to better address the psychological dimensions of dentophobia, thereby improving patient compliance and long-term oral health outcomes. When incorporated within an integrative care framework—alongside established methods such as tell–show–do, cognitive-behavioral techniques, or minimal pharmacological support—VRET may offer a more comprehensive and patient-centered strategy for reducing dental fear, ultimately fostering greater trust and cooperation between patients and providers.

Future studies should further explore the integration of VRET in diverse clinical contexts. One important direction is the inclusion of broader and more heterogeneous populations, such as older adults and individuals with limited digital literacy, to better understand the generalizability of findings. In addition, cross-cultural research is needed, as cultural norms strongly influence how patients perceive both dental care and technology-based interventions.

Technological innovation also offers multiple avenues for advancement. The development of adaptive VR scenarios, guided by real-time biometric feedback (e.g., heart rate variability, galvanic skin response), could allow for greater personalization and more effective anxiety reduction. Incorporating artificial intelligence–driven algorithms into VRET platforms may further optimize exposure intensity and duration, tailoring therapy to individual patient needs.

At the same time, mechanistic studies should be encouraged, using neuroimaging or psychophysiological methods to clarify how VRET produces its therapeutic effects. Such insights would help bridge the gap between clinical observations and underlying biological processes.

In addition, future studies should also assess patients’ subjective feelings and fear levels in scenarios where no actual dental procedures are performed, in order to further validate the role of VR in modulating dental anxiety.

Finally, future work should evaluate the scalability and cost-effectiveness of VRET in routine dental practice, as well as its acceptance among dental professionals. Implementation studies, pilot programs, and health-economic analyses will be crucial in determining whether VRET can move from experimental settings into standard care pathways.

## 5. Conclusions

This study provides evidence that Virtual Reality Exposure Therapy (VRET) can serve as an effective and clinically relevant tool in the management of dentophobia. Beyond reducing anxiety scores in the short term, VRET demonstrated sustained benefits over time, improving patients’ willingness to attend dental appointments and decreasing avoidance behaviors. These outcomes highlight the potential of VRET to complement or enhance existing behavioral management techniques, such as the tell–show–do method, by offering a more immersive and engaging form of exposure.

From a practical perspective, integrating VRET into current dental protocols could improve patient cooperation, reduce treatment delays caused by avoidance, and ultimately contribute to better oral health outcomes. The acceptability and feasibility observed in this study also suggest that VRET can be realistically implemented in clinical settings, particularly for younger and digitally fluent populations. As digital therapeutics gain momentum in healthcare, VRET offers a forward-looking approach that can modernize dentophobia management and expand the range of evidence-based tools available to dental practitioners.

## Figures and Tables

**Table 1:** MDAS score summary across time points for both groups.

Time Point	VRET Mean ± SD	Traditional Mean ± SD
Baseline	15.8 ± 1.2	16.0 ± 0.9
Post-Intervention	8.8 ± 1.2	8.5 ± 1.5
1 Week	9.0 ± 1.3	9.2 ± 1.4
3 Months	9.0 ± 1.3	9.2 ± 1.4
6 Months	9.0 ± 1.5	10.5 ± 1.7

**Table 2:** Participant satisfaction and preferences.

Measure	VRET Group	Traditional Group
Satisfaction (%)	86%	64%
Perceived Control (Mean ± SD)	8.2 ± 1.1	6.7 ± 1.4
Preference for Method (%)	79%	55%

## Data Availability

Datasets used in this study are available upon request.
